# Enrichment Map – a Cytoscape app to visualize and explore OMICs pathway enrichment results

**DOI:** 10.12688/f1000research.4536.1

**Published:** 2014-07-01

**Authors:** Ruth Isserlin, Daniele Merico, Veronique Voisin, Gary D. Bader

**Affiliations:** 1 The Donnelly Centre, University of Toronto, Toronto, ON M5S 3E1, Canada; 2 The Centre for Applied Genomics, Program in Genetics and Genome Biology, The Hospital for Sick Children, Toronto, ON M5G 1X8, Canada

## Abstract

High-throughput OMICs experiments generate signals for millions of entities (i.e. genes, proteins, metabolites or any measurable biological entity) in the cell. In an effort to summarize and explore these signals, expression results are examined in the context of known pathways and processes, through enrichment analysis to generate a set of pathways and processes that is significantly enriched. Due to the high redundancy in annotation resources this often results in hundreds of sets. To facilitate the analysis of these results, we have developed the Enrichment Map app to visualize enrichments as a network. We have updated Enrichment Map to support Cytoscape 3, and have added additional features including new data formats and command line access.

## Introduction

With the expansion and accessibility of a wide range of experimental techniques to accurately identify and measure any known genomics feature ranging from proteins, transcripts, genes, microRNAs, copy number variations, or DNA methylation in a high-throughput manner, signals for thousands of entities are often generated for an individual OMICs experiment. In efforts to interpret these results in the context of perturbed cellular mechanisms, the entities are often scored and examined for enrichment in known pathways and processes.

Pathway enrichment analysis helps to uncover general trends or themes present in the data, instead of focusing on one or a few favorite differential genes. Available tools are abundant, designed for varying data types and implemented using a range of different statistical tests: given a set of biological entities, these OMICs signals are then translated into a set of significant pathways and processes (reviewed in Khatri *et al.* ^1^, Huang *et al.* ^2^). Due to the high redundancy that exists between pathway databases coming from multiple functional annotations of gene products, pathway enrichment often results in a long list of potentially interesting pathways. To help analyze the set of differential pathways, we created the Enrichment Map app to display enrichment results as a network, where pathways are nodes in the network and edges represent known pathway cross-talk defined by the number of genes shared between the pair of pathways and where the network layout organizes the map into functional modules ^3^.

In this paper, we present the recent implementation of the Enrichment Map app for Cytoscape 3 as well as new features.

## Implementation

Although originally designed to support Gene Set Enrichment Analysis (GSEA) ^4^ the current Enrichment Map app supports multiple enrichment results from tools such as DAVID ^5^, BiNGO ^6^, and GREAT ^7^ as well as simplified generic input files which one can easily create from your own enrichment results. Tools like g:Profiler ^8^ allow users to download results in an Enrichment Map compatible generic format.

With the ongoing effort to populate gene annotation and pathway databases, it is difficult for standalone enrichment tools to keep databases up to date. For convenience, we compile gene set files or GMT files, a format created for the GSEA software, to describe all the genes contained in a specified gene set, monthly, from a comprehensive set of annotation and Pathway databases ( http://download.baderlab.org/EM_Genesets/), including standard sources, like MSigDB ^4^. Although originally GMT files were specific to GSEA, with the expansion of R and Bioconductor it is now straightforward to load GMT files into data structures in R using packages like GSA ( http://statweb.stanford.edu/~tibs/ftp/GSA.pdf) and analyze your OMICs expression data with one of the many different gene set enrichment algorithms such as geneSetTest in the Limma package ^9^, global test ^10^, or Camera ^11^. Visualizing the resulting enrichments is straightforward by exporting to our generic format which minimally consists of the geneset name, description and associated enrichment p-value. Through this mechanism, no matter what the dataset of interest is, gene, protein or metabolite expression, the resulting enrichment analysis can be displayed as an enrichment map.

**Figure 1.**
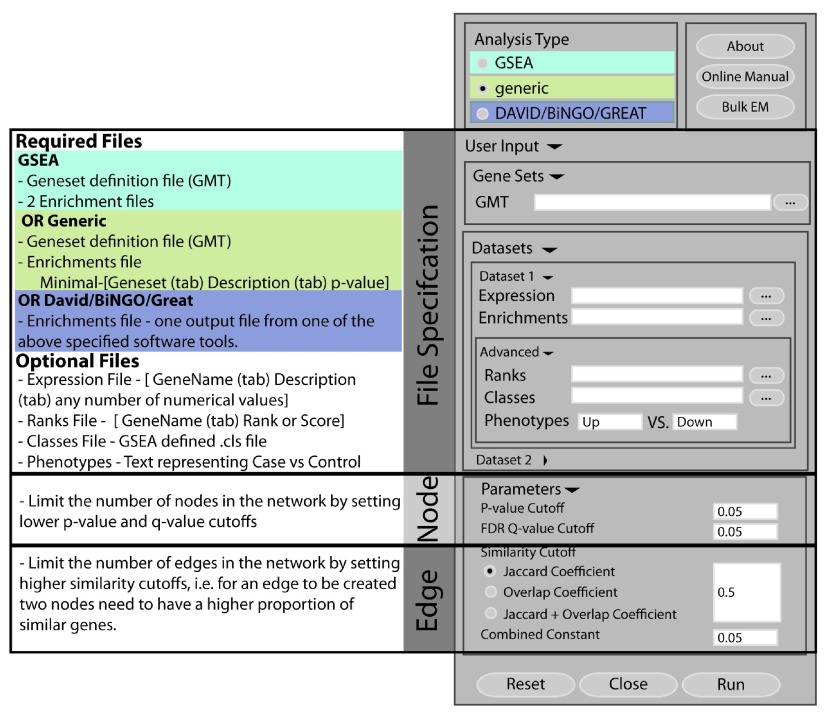
Enrichment Map app user interface Illustration of Enrichment Map user interface which consists of four main parts: analysis type, file specifications, node and edge filtering. For each analysis type there is a different set of required files. For added functionality there are a set of optional files that can be included to help annotate and explore results. Tuning parameters such as p-value and q-value helps control the number of nodes while tuning the similarity coefficient helps control the number of edges.

There are two main ways to input data into Enrichment Map, through the user interface ( Figure 1) or the command tool ( Table 1). The user interface is an interactive way to specify all the required files and parameters based on the analysis type chosen. The command tool allows users to automatically create maps directly from the command line, other Cytoscape apps or other programs which can include in-house enrichment tools.

**Table 1.** Command tool specification outlined for each of the analysis types. There is an additional command optimized for GSEA inputs only.

Command	Required Arguments	Optional Arguments
enrichment map build analysistype="GSEA"	**gmtFile**=filepath to geneset file **enrichmentsDataset1**=filepath to enrichments **enrichments2Dataset1**=filepath to enrichments **pvalue**=numerical cutoff, {default : 0.05} **qvalue**=numerical cutoff, {default : 0.1} **coefficients**=one of the following [OVERLAP, JACCARD, COMBINED], {default:OVERLAP} **similaritycutoff**=numerical cutoff, {default : 0.5}	**expressionDataset** 1=filepath to expression file **ranksDataset** 1=filepath to rank file **classDataset** 1=filepath to class file **phenotype1Dataset** 1=Text representing Phenotype **phenotype2Dataset** 1=Text representing Phenotype2 **enrichmentsDataset2**=filepath to enrichments **enrichments2Dataset2**=filepath to enrichments (Replace 1 for 2 to specify which dataset the file is)
enrichmentmap build analysistype="generic"	**gmtFile**=filepath to geneset file **enrichmentsDataset** 1=filepath to enrichments **pvalue**=numerical cutoff, {default : 0.05} **qvalue**=numerical cutoff, {default : 0.1} **coefficients**=one of the following [OVERLAP, JACCARD, COMBINED], {default:OVERLAP} **similaritycutoff**=numerical cutoff, {default : 0.5}	**expressionDataset** 1=filepath to expression file **ranksDataset** 1=filepath to rank file **classDataset** 1=filepath to class file **phenotype1Dataset** 1=Text representing Phenotype **phenotype2Dataset** 1=Text representing Phenotype2 **enrichmentsDataset2**=filepath to enrichments (Replace 1 for 2 to specify which dataset the file is)
enrichmentmap build analysistype= "David/BiNGO/Great"	**enrichmentsDataset1**=filepath to enrichments **pvalue**=numerical cutoff, {default : 0.05} **qvalue**=numerical cutoff, {default : 0.1} **coefficients**=one of the following [OVERLAP, JACCARD, COMBINED], {default:OVERLAP} **similaritycutoff**=numerical cutoff, {default : 0.5}	**expressionDataset** 1=filepath to expression file **enrichmentsDataset2**=filepath to enrichments (Replace 1 for 2 to specify which dataset the file is)
enrichmentmap gseabuild	**edb**=filepath to GSEA results edb directory **pvalue**=numerical cutoff, {default : 0.05} **qvalue**=numerical cutoff, {default : 0.1} **coefficients**=one of the following [OVERLAP, JACCARD, COMBINED], {default:OVERLAP} **similaritycutoff**=numerical cutoff, {default : 0.5}	**expression**=filepath to expression file **expression2**=filepath to expression file **edbdir2**=filepath to edb directory

**Figure 2.**
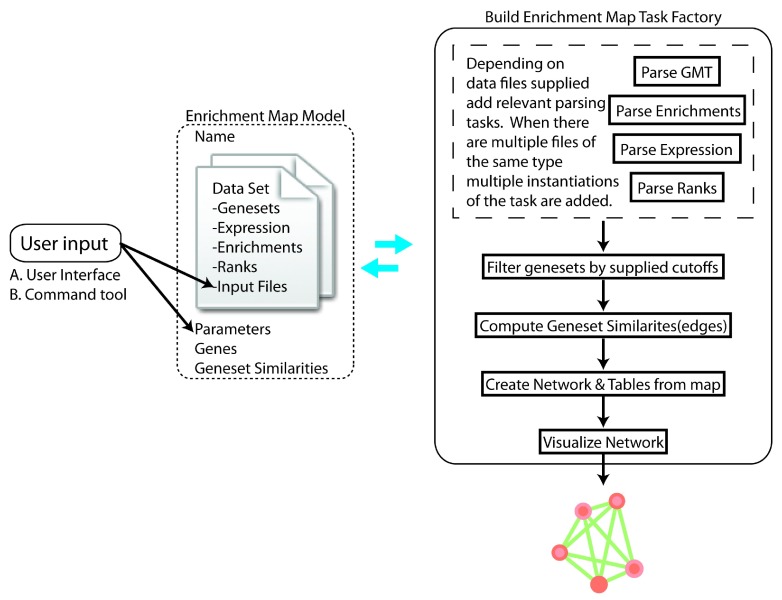
Enrichment Map build process overview.

Once files and parameters have been specified, the Enrichment Map can be created. Unlike a traditional biological network, nodes in an Enrichment Map represent a set of genes (e.g. a pathway) and their connections the set of genes that two nodes have in common (e.g. pathway cross-talk). Every Enrichment Map is associated with a set of files, parameters, and a number of datasets (currently limited to two) ( Figure 2). Datasets contain gene sets, enrichments, and expression all of which is needed to interactively update the map through cutoff adjustment sliders found in the legend panel or display the genes contained in a given node or edge selection as a heatmap.

Enrichment Map app was ported to Cytoscape 3 as a bundle app using Open Service Gateway initiative (OSGi) services provided through the extensive Cytoscape API (version 3.1). The look and feel of the app remains similar to the original implementation for Cytoscape 2 with user input interfaces and view panels including expression heatmap and legend being a direct port from the original source. Given the new framework, each panel implements the CytoPanelComponent and is a registered service associated with the Enrichment Map app. The main enrichment map input panel is registered only once a user opens the app. The remaining view panels are only registered once an enrichment map is created. Enrichment Map consists of one main taskFactory that given an Enrichment Map object populated with a set of input files will construct the appropriate task iterator. Depending on the files specified different parsing tasks can be added to the iterator. Additionally, multiple files of the same type can also be added to the queue with distinct instantiations of a parsing task (with different files specified on task creation). All parsed files populate fields contained in the Enrichment Map object which is then passed to and updated by each of the subsequent tasks ( Figure 2).

The BuildEnrichmentMapTaskFactory is used by both the user interface and command tool to construct an enrichment map. Command tool functionality for Enrichment Map requires the given task to define its variables as tunables. Tunables are user supplied information needed by the task. User interfaces can be automatically generated for such tasks based on the set of tunable definitions. When implementing the Enrichment Map tunable task it was our intention to replace our current user interface with the one automatically generated by the task. Given the varied data required from the user as well as the interactive nature of our current user interface the generated tunable interface although functional lacked features that our users are accustomed to. For instance, to specify the analysis type or similarity cutoff our interface has two sets of radio buttons where all the options are visible and only one is selectable. In the tunable interface the same choice can only be represented as a single selection list, a drop down list the user can choose one option from. Both representations are functional but we preferred the radio button implementation therefore, we decided to keep our original interface and add the tunable task solely for the command tool functionality.

## Results

**Figure 3.**
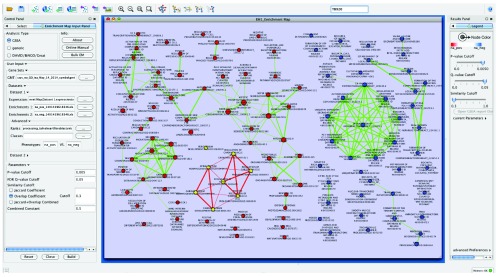
Enrichment Map of heart fibroblast versus tail fibroblast expression. Using the search field you can enter any text to search all attributes of the given network. Highlighted nodes, (shown as yellow nodes with red edges just left of center) are genesets that contain the gene TBX20.

To illustrate the functionality of Enrichment Map we analyzed and visualized an expression dataset from the Gene Expression Omnibus (GEO) ^12^ for mouse fibroblast cells. The experiment was designed to compare gene expression in fibroblast cells in the heart to those in the tail to highlight genes that are uniquely expressed in heart fibroblasts ^13^ (GSE50531). Raw expression data was scored using the GEO2R tool available on the GEO website. These expression data were input to GSEA along with a recent compilation of mouse pathway gene sets (May 14, 2014; http://download.baderlab.org/EM_Genesets/May_14_2014/) to calculate enrichments. GSEA output files were given to the app with the cutoffs p-value < 0.005, q-value < 0.05 and overlap similarity coefficient > 0.3. The Enrichment Map generated had roughly the same number of enriched gene sets specific to heart as to tail with cardiac specific sets associated only with the heart phenotype ( Figure 3, red nodes).

**Figure 4.**
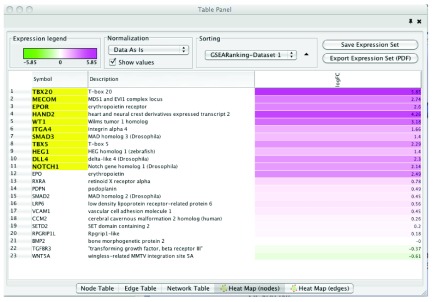
Node Heat Map Panel (contained in the Cytoscape table panel) displayed on selection of “Pericardium development (GO:0060039)” gene set. If GSEA results are loaded into Enrichment Map, GSEA leading edge genes, defined as the set of genes that contribute most to the enrichment, are highlighted in yellow.

One of the main genes mentioned in the paper associated with this dataset was TBX20 as a specific cardiogenic fibroblast gene found to be important for both normal cardiac development and postinfarct repair ^13^. In Enrichment Map it is easy to find all gene sets that contain it by entering the term TBX20 into the search box ( Figure 3) (this will also highlight any gene sets that have TBX20 in the name or any other attribute). Built-in search functionality in Cytoscape 3 has improved from Cytoscape 2. All attributes associated with a given network are indexed so there is no longer the need to specify which attribute you would like to search through. Selection of individual or sets of nodes and edges creates a view of the genes contained within the selection as a heat map ( Figure 4).

Often one of the main challenges after creating an Enrichment Map is going from a network in Cytoscape to publication quality figures. We format the labels so they are more readable and don’t extend across the whole screen, but as a result modules often contain overlapping labels that are difficult to read and require hours of manual formatting to create networks that can be used for figures. Using the Cytoscape 3 built-in scaling feature (Layout>Scale), the visualization of clusters and networks can be improved.

## Conclusions

The Enrichment Map app allows users to translate large sets of enrichment results to a network where highly similar terms cluster together to better highlight overall trends and themes of the underlying data. The details behind the enrichment can be further investigated within the Enrichment Map app using the built-in expression viewer to see all the entities associated with a selected pathway.

## Software availability

Software available from: http://apps.cytoscape.org/apps/enrichmentmap

Latest source code: https://github.com/BaderLab/EnrichmentMapApp

Source code as at the time of publication: https://github.com/F1000Research/EnrichmentMapApp/releases/tag/V1.0

Archived source code as at the time of publication: http://dx.doi.org/10.5281/zenodo.10542 ^14^

License: Lesser GNU Public License 2.1: https://www.gnu.org/licenses/old-licenses/lgpl-2.1.html

Tutorials http://baderlab.org/Software/EnrichmentMap#Tutorials_and_Examples
