# Supplementary material for: Enrichment Map – a Cytoscape app to visualize and explore OMICs pathway enrichment results
Source: F1000Res. 2014 Jul 1;3:141. [Version 1] doi: 10.12688/f1000research.4536.1 (PMC4103489; doi:10.12688/f1000research.4536.1)
Supplement: Peer review history

# Peer review: Referee response for version 1

**DOI:** 10.5256/f1000research.4852.r5298

**Published:** 2014-07-15

**Authors:** Florian Markowetz

**Affiliations:** 1 Department of Oncology, University of Cambridge, Cambridge, UK

This paper is an update on the Enrichment Map introduced in 2010 by the same authors. They have extended the methodology and made it available in the newest version of Cytoscape.

Gene set enrichment methods of various forms are one of the most widely used first steps to gain a global picture of which pathways or other functional units are involved in some molecular phenotype. However, it is generally very hard to make sense of the results - mostly because lists of enriched gene sets can be very long and might be due to a small set of genes that appear in many of them.

This is where the Enrichment Map comes in: By making the overlap between gene sets explicit it allows users to visually explore 'enrichment themes' (clusters of overlapping gene sets). Because it is simple and informative I believe this will become the standard way to present enrichment results.

I have read this submission. I believe that I have an appropriate level of expertise to confirm that it is of an acceptable scientific standard.

# Peer review: Referee response for version 1

**DOI:** 10.5256/f1000research.4852.r5301

**Published:** 2014-07-14

**Authors:** Nathan Salomonis

**Affiliations:** 1 Division of Biomedical Informatics, Cincinnati Children's Hospital Medical Center Research Foundation, Cincinnati, OH, USA

The authors present an already highly used and very useful approach for making sense of highly redundant biological enrichment results that arise from the analysis of transcriptome and genomics datasets. The added additional utility of accessing the software by command line and using alternative input formats makes this a highly accessible plugin for Cytoscape, that will clearly be widely adopted by the Cytoscape community through its availability in Cytoscape 3.0.

I have read this submission. I believe that I have an appropriate level of expertise to confirm that it is of an acceptable scientific standard.
